# Selenoprotein-P1 (SEPP1) Expression in Human Proximal Tubule Cells after Ischemia-Reperfusion Injury: An In Vitro Model

**DOI:** 10.3390/medicina60060875

**Published:** 2024-05-27

**Authors:** Giuseppe Coppolino, Marilena Celano, Michela Musolino, Mario D’Agostino, Mariateresa Zicarelli, Michele Andreucci, Carmen De Caro, Diego Russo, Emilio Russo, Davide Bolignano

**Affiliations:** 1 Nephrology and Dialysis Unit, Magna Graecia University Hospital, 88100 Catanzaro, Italy; 2 Department of Health Sciences, Magna Graecia University, 88100 Catanzaro, Italy; 3 Department of Pharmacy, University of Naples “Federico II”, 80131 Naples, Italy; 4 Department of Medical and Surgical Sciences, Magna Graecia University, 88100 Catanzaro, Italy

**Keywords:** selenoprotein-p1, SEPP1, sodium selenite, acute kidney injury; selenium, HK-2 cells, renal ischemia-reperfusion (I/R) injury, human tubular cells

## Abstract

*Background and Objectives*: Selenium deficiency represents a risk factor for the occurrence of severe diseases, such as acute kidney injury (AKI). Recently, selenoprotein-p1 (SEPP1), a selenium transporter, mainly released by the liver, has emerged as a promising plasmatic biomarker of AKI as a consequence of cardio-surgery operations. The aim of the present study was to investigate, on an in vitro model of hypoxia induced in renal tubular cells, HK-2, the effects of sodium selenite (Na_2_SeO_3_) and to evaluate the expression of SEPP1 as a marker of injury. *Materials and Methods*: HK-2 cells were pre-incubated with 100 nM Na_2_SeO_3_ for 24 h, and then, treated for 24 h with CoCl_2_ (500 µM), a chemical hypoxia inducer. The results were derived from an ROS assay, MTT, and Western blot analysis. *Results*: The pre-treatment determined an increase in cells’ viability and a reduction in reactive oxygen species (ROS), as shown by MTT and the ROS assay. Moreover, by Western blot an increase in SEPP1 expression was observed after hypoxic injury as after adding sodium selenite. *Conclusions*: Our preliminary results shed light on the possible role of selenium supplementation as a means to prevent oxidative damage and to increase SEPP1 after acute kidney injury. In our in vitro model, SEPP1 emerges as a promising biomarker of kidney injury, although further studies in vivo are necessary to validate our findings.

## 1. Introduction

Renal ischemic/reperfusion (I/R) injury is a serious complication of acute kidney injury (AKI), the consequence of an insufficient blood supply to the kidneys. It firstly involves a compromised delivery of oxygen and nutrients to renal tissues during the ischemic phase. The restoration of blood flow during reperfusion paradoxically exacerbates the damage, causing oxidative stress, inflammation, and additional cellular injury [1,2,3]. Some typical situations occur during severe dehydration as a consequence of major surgery or in the setting of cardiovascular emergencies. In cardiac surgery, the need for the use of cardiopulmonary bypass (CPB) is associated with an extremely high risk of AKI with a post-operative incidence peak that can even reach 20–30% [4]. This condition amplifies the risk of post-operative morbidity and mortality because it is correlated with the occurrence of complications such as heart failure and stroke. Despite the development of less invasive techniques, cardiac surgery remains the first option in many conditions such as severe coronary artery disease, valve diseases, and complex interventions [5]. If specific well-timed therapeutic strategies are not adopted AKI can evolve in chronic kidney disease, increasing healthcare costs [6]. Novel biomarkers, anticipating serum creatinine rise, are needed to have a speedy diagnosis of acute kidney injury, and thus, to mitigate and to preserve kidney function with well-timed therapeutic plans. Cystatin C, kidney injury molecule-1, and human neutrophil gelatinase-associated lipocalin have been found to change earlier than creatinine, particularly when measured in combination, so their use in clinical practice can facilitate early diagnosis and treatment of AKI [7]. The urgency for new markers stems from the critical need to swiftly diagnose AKI and implement timely therapeutic interventions to preserve renal function [8]. AKI represents a significant clinical challenge, characterized by a sudden decline in kidney function over a short period, often resulting from various etiologies such as ischemia, nephrotoxicity, or sepsis. While serum creatinine remains the cornerstone for assessing renal function, its limitations in detecting the early stages of AKI and its delayed responsiveness underscore the necessity to find alternative biomarkers. Recently, in our pilot study on patients undergoing elective major cardiac surgery with cardiopulmonary bypass (CPB), selenoprotein-p1 (SEPP1) has emerged as a promising candidate plasmatic biomarker for an early AKI risk stratification [9]. SEPP1 acts as a selenium transporter, supplying tissues and organs with this trace mineral. Selenium is an essential trace micronutrient, being a component of the amino acids selenocysteine and selenomethionine. It plays a critical role in various physiological processes within the human body. Selenium is a vital trace element necessary for the biosynthesis of a specific group of proteins known as selenoproteins, which contain the amino acid selenocysteine and that play crucial roles in controlling key metabolic pathways in eukaryotes [10,11,12,13]. As an integral component of selenoproteins, selenium contributes to antioxidant defense, thyroid hormone metabolism, immune function, and reproductive health. The distribution of selenium in tissues and organs is tightly regulated to ensure optimal functioning of these processes. The biosynthesis of selenoproteins is meticulously regulated in a hierarchical manner, ensuring that essential selenium-dependent processes are maintained even in the face of severe selenium deficiency. These selenoproteins serve diverse functions such as regulating thyroid hormone levels, protecting against oxidative stress, bolstering the immune response, participating in redox-related signaling, and overseeing the quality control of secreted or aged proteins [14,15]. The intricate nature of selenium biology prompts the use of systems biology techniques, including metabolomics, genomics, and proteomics, to unravel its role. In a recent study, a global proteomic approach was employed in a clinical trial where patients received either selenized-yeast or placebo-yeast supplementation for 48 weeks. This analysis revealed significant alterations in the expression of plasma proteins associated with different metabolic pathways such as immune functions, lipid metabolism, and insulin resistance following selenium supplementation. These findings underscore the potential of omics technologies in elucidating selenium-responsive markers and selenium’s impact on various physiological pathways [16].

A deficiency in selenium elevates the risk of various common human ailments including cancer, infections, cognitive decline, cerebrovascular events, and thyroid disorders. Notably, several experimental studies and clinical trials have shown promising outcomes with selenium supplementation in conditions such as severe sepsis, autoimmune thyroid disease, stroke, and cardiac arrest [17,18,19]. However, the intricate interplay between selenium status, selenoproteins, and these diseases poses a challenge given the diverse underlying mechanisms and molecular pathways involved [20]. We reasoned that an insufficient perfusion of affected tissues may represent a common underlying motif of this set of apparently unrelated pathologies. Hypoxia might constitute a common stimulus for adapting Se metabolism and selenoprotein expression to the actual needs of the cells under low oxygen supply. SEPP1 is primarily secreted from the liver but it is also expressed in other tissues [21,22] such as kidney and heart tissue, expressing high concentrations of mRNA, and less in lung, skeletal muscle, testis, and brain [14,23]. Understanding the role of selenium supply in tissues and organs provides valuable insights into its physiological significance and potential implications for health and disease. Different animal models are usually used in the study of renal (I/R) injury but we conversely investigated some renal effects at the cellular level with the benefit of having cells with the same biological properties, excluding individual characteristics. The aims of this study were to investigate, in an in vitro model of hypoxic renal injury, the response of wounded tubular cells to selenium supply and the expression of SEPP1 as marker of renal tubular cell injury.

## 2. Materials and Methods

### 2.1. Cell Culture

In this study immortalized proximal tubular epithelial cell lines (HK-2) were used. The cells were purchased from American Type Culture Collection (LGC Standards s.r.l., Milan, Italy). The HK-2 cells were cultured in Keratinocyte-SFM, a complete serum-free medium supplemented with human recombinant epidermal growth factor (rEGF) and bovine pituitary extract (BPE) (LGC Standards s.r.l). The medium was supplemented with penicillin (100 UI/mL), streptomycin (0.1 mg/mL), and amphotericin B (2.5 µg/mL) (Sigma Aldrich s.r.l., Milan, Italy). The cells were cultured at 37 °C in a humidified 5% CO_2_ atmosphere.

### 2.2. Cell Viability

Cell proliferation was evaluated by MTT assay. The experimental design included three treatments: 1—cells treated with sodium selenite (Sigma Aldrich S.r.l., Milan, Italy); 2—cells treated with cobalt chloride (CoCl_2_) (Sigma Aldrich s.r.l., Milan, Italy); 3—cells pre-treated for 24 h with sodium selenite, and then, treated with CoCl_2_ for another 24 h. In brief, HK-2 cells were seeded in 96-well plates at density of 6 × 10^3^, and after 24 h the cells were pre-treated or not with fresh medium containing 100 nM sodium selenite (Na_2_SeO_3_) for 24 h. The next day, HK-2 cells were exposed or not to CoCl_2_ (a known chemical hypoxia inducer) [24,25] for an additional 24 h at 500 µM. At the end of the incubation period the medium was replaced with fresh medium containing methyl thiazolyl tetrazolium (MTT). After 3 h, the solubilized formazan product was quantified with a microplate spectrophotometer (VARIOSKAN LUX, Thermo Fisher Scientific Inc., Waltham, MA, USA) at a wavelength of 540 nm and a reference wavelength of 690 nm. Results are expressed as percentages over untreated cells. Cell mortality was evaluated by trypan blue dye exclusion assay. Briefly, experiments were carried out in 12-well plates containing 50 × 10^3^ cells. After incubation with Na_2_SeO_3_ and CoCl_2_ as previously described, cells were trypsinized and the pellet was resuspended in trypan blue buffer (0.4%). The cells were counted by hemocytometer chamber. Cell mortality was calculated as the percentage of stained cells over the total cell number and expressed as the ratio between treated and untreated cells.

### 2.3. Protein Extraction and Western Blot Analysis

After treatment with Na_2_SeO_3_ and CoCl_2_, as previously described, proteins were extracted [26]. A mass of 20 µg of proteins was run on a 12% SDS-PAGE gel, transferred to PVDF membranes (VWR, Milan, Italy), blocked with phosphate-buffered saline, 0.1% Triton, and 5% non-fat dry milk (PBS-T/milk), and incubated overnight with the following antibodies: anti-SEPP1 diluted 1:250 (Abcam, Cambridge, UK) and anti-α-tubulin diluted 1:10,000, used as an internal control. Then, the membranes were washed in PBS-T and incubated with horseradish peroxidase-conjugated anti-rabbit or anti-mouse antibody (Transduction Laboratories, Lexington, KY, USA) in PBS-T. The Western blot detection system ECL Plus (Perkin Elmer, Monza, Italy) was used to visualize proteins.

### 2.4. Flow Cytometer Analysis/ROS Assay

After incubation of cells with Na_2_SeO_3_ and CoCl_2_ as earlier mentioned, cellular ROS production was measured [27]. Briefly, HK-2 cells were pre-treated or not for 24 h with 100 nM Na_2_SeO_3_, and then, exposed for 24 h to 500 µM CoCl_2_. After that, the cells were trypsinized and resuspended in medium containing 25 µM 2-7-Dichlorodihydro-fluorescein diacetate (H2DCF-DA), a fluorescent probe, for 30 min in the dark, at 37 °C. Then, the cells were centrifuged and the pellet resuspended in PBS. The fluorescence was evaluated by flow cytometry (FACS Canto II, Becton Dickinson, San Jose, CA, USA) and 200 µM hydrogen peroxide (H_2_O_2_) was used as an internal positive control.

### 2.5. Statistical Analysis

The results were analyzed by one-way ANOVA followed by the Tukey–Kramer multiple comparisons test. All results are expressed as mean ± standard error of mean (SEM) and *p* values lower than 0.05 are considered statistically significant. All statistical analyses were performed using the GraphPad Prism version 5.0 statistical software (GraphPad Software Inc., San Diego, CA, USA).

## 3. Results

### 3.1. Effects of CoCl_2_ and Sodium Selenite on Cell Viability and Cellular Morphology

**Figure 1:**
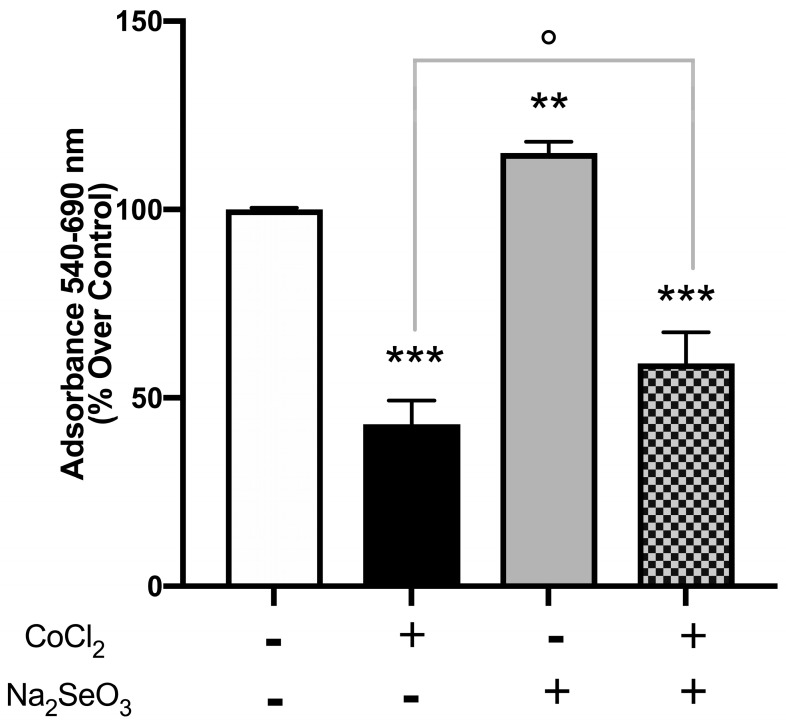
Effects of Na_2_SeO_3_ and CoCl_2_ on cell viability. After hypoxia induced by CoCl_2_, cell growth was evaluated by MTT assay as described in Materials and Methods. Results are expressed as percentage of cell viability vs. untreated cells and represent means ± SEM from at least three independent experiments. White column: without CoCl_2_ and Na_2_SeO_3_; Black column: with CoCl_2_ and without Na_2_SeO_3_; Gray column without CoCl_2_ and with Na_2_SeO_3_; Checkered pattern: with CoCl_2_ and Na_2_SeO_3_. Statistical analysis was performed by using the Tukey–Kramer multiple comparisons test. *** *p*< 0.001 vs. untreated cells; ** *p* < 0.01 vs. untreated cells; ° *p* < 0.001 vs. CoCl_2_.

Renal tubular cells represent a key target in acute kidney injury caused by I/R and in general during hypoxic conditions. We performed our experiments on immortalized proximal tubular cells, HK-2, derived from a normal human adult male kidney exposed or not to 500 μM CoCl_2_, as already reported in the literature, sufficient to induce hypoxic conditions [24,25]. Moreover, the protective effect of sodium selenite (the inorganic form of selenium) on the hypoxic conditions was evaluated. As shown in Figure 1, the treatment for 24 h with CoCl_2_ alone determined an inhibitory effect on cell survival (about 60%; *p* < 0.001 vs. control), while exposure for 24 h to sodium selenite without other stimuli resulted in an increase in cell growth (about 20%; *p* < 0.01 vs. control) with respect to untreated cells. When cells were pre-treated for 24 h with sodium selenite, it was interesting to observe a reduction in the CoCl_2_ induced effect.

**Figure 2:**
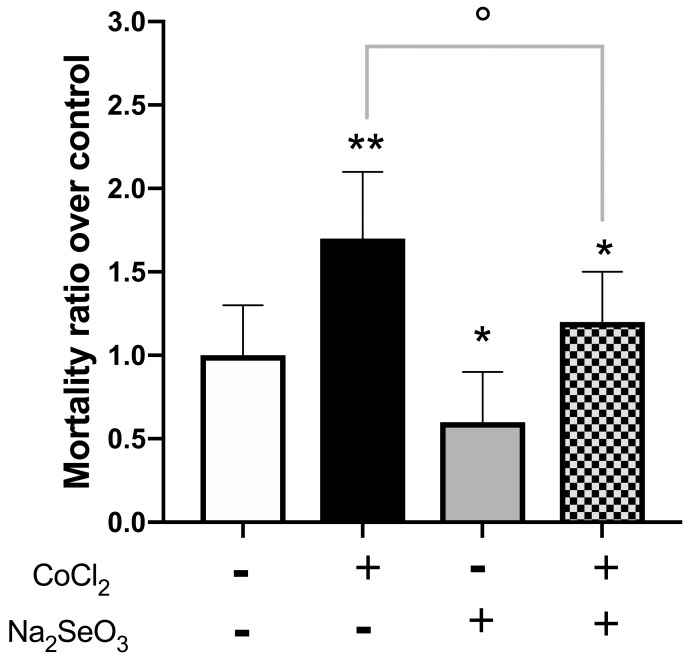
Cytotoxic effects of Na_2_SeO_3_ and CoCl_2_. Cell mortality was evaluated by trypan blue exclusion, as described in Materials and Methods. The values were expressed as the ratio over untreated cells and represent means ± SEM of three independent experiments. White column: without CoCl_2_ and Na_2_SeO_3_; Black column: with CoCl_2_ and without Na_2_SeO_3_; Gray column without CoCl_2_ and with Na_2_SeO_3_; Checkered pattern: with CoCl_2_ and Na_2_SeO_3_. Statistical analysis was performed by using the Tukey–Kramer multiple comparisons test. ** *p* < 0.01 vs. untreated cells; * *p* < 0.05 vs. untreated cells. ° *p* < 0.001 vs. CoCl_2_.

In fact, as shown in Figure 1 the inhibition of proliferation was significantly reduced (about 40%; *p* < 0.001 vs. control and about 15% CoCl_2_ vs. CoCl_2_ + Na_2_SeO_3_ (*p* < 0.05). Moreover, in the same conditions, cell mortality was evaluated by trypan blue dye exclusion assay. As shown in Figure 2, the pre-treatment with sodium selenite reduced the effect on cell mortality observed with CoCl_2_ alone with respect to the control (*p* < 0.01) but also comparing CoCl_2_ vs. CoCl_2_ + Na_2_SeO_3_ (*p* < 0.01).

**Figure 3:**
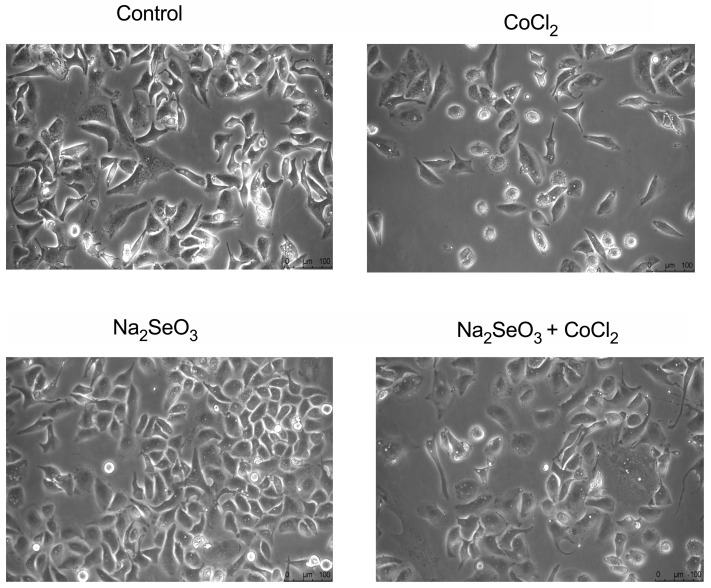
Effects of Na_2_SeO_3_ and CoCl_2_ on HK-2 cells’ morphology. Cell morphological observation was performed using a LEICA microscope (DMIL LED) after treatment of HK-2 cells with CoCl_2_ (500 µM) alone or after pre-treatment with Na_2_SeO_3_ (100 nM) (magnification 20×). Control indicates untreated cells.

In addition, analyzing the effects of chemical hypoxia on HK-2 cells, we found that CoCl_2_ alone determined a change in cell morphology (Figure 3), while this effect was less evident when the cells were pre-treated with sodium selenite. In terms of cellular morphology, cells treated with CoCl_2_ tended to shrink and to die. Our data showed that the treatment with sodium selenite protects against the loss of cell proliferation and of morphological cellular change induced by CoCl_2_.

### 3.2. Effects on ROS Production after Exposure to CoCl_2_ and/or Sodium Selenite

**Figure 4:**
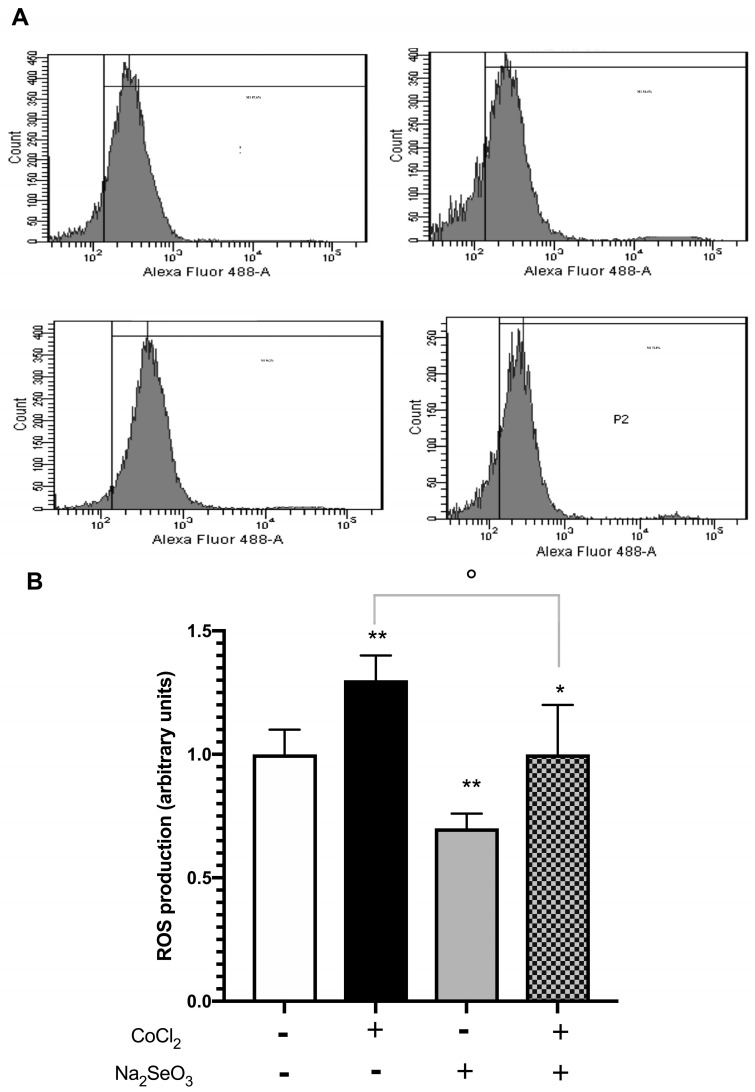
ROS levels in HK-2 cells treated with Na_2_SeO_3_ and CoCl_2_. HK-2 cells were pre-treated with Na_2_SeO_3_ (100 nM) for 24 h, and then, exposed to CoCl_2_ (500 µM) for 24 h. The fluorescence of DCF was evaluated by flow cytometry after incubation with H2DCF-DA, as described in Materials and Methods. Panel (**A**): cytofluorimetric plots representative of ROS intracellular level. Panel (**B**): the data show the ROS production expressed as fold of increase with respect to untreated cells, considered arbitrarily as 1. The results represent the mean ± SEM of three independent experiments. White column: without CoCl_2_ and Na_2_SeO_3_; Black column: with CoCl_2_ and without Na_2_SeO_3_; Gray column without CoCl_2_ and with Na_2_SeO_3_; Checkered pattern: with CoCl_2_ and Na_2_SeO_3_. Statistical analysis was performed using the Tukey–Kramer multiple comparisons test. ** *p* < 0.01 vs. untreated cells; * *p* < 0.05 vs. untreated cells. ° *p* < 0.001 vs. CoCl_2_.

AKI can induce ROS generation [28,29,30], and for this reason, to confirm the protective role of pre-treatment with sodium selenite previously observed, ROS production was evaluated by fluorescence-activated cell sorting (FACS). As shown in Figure 4B, treatment with CoCl_2_ enhanced intracellular ROS levels with respect to the control (*p* < 0.01) while incubation with sodium selenite decreased ROS levels when compared to untreated cells (*p* < 0.01). In cells pre-treated with sodium selenite before cobalt chloride, instead, a reduction in ROS levels was found compared to CoCl_2_-treated cells (*p* < 0.05). As shown in Figure 4A, the attenuated effect on ROS production observed in pre-treated cells was revealed by a leftward shift in fluorescence.

### 3.3. Effects of CoCl_2_ and Sodium Selenite on the Expression of SEPP1

**Figure 5:**
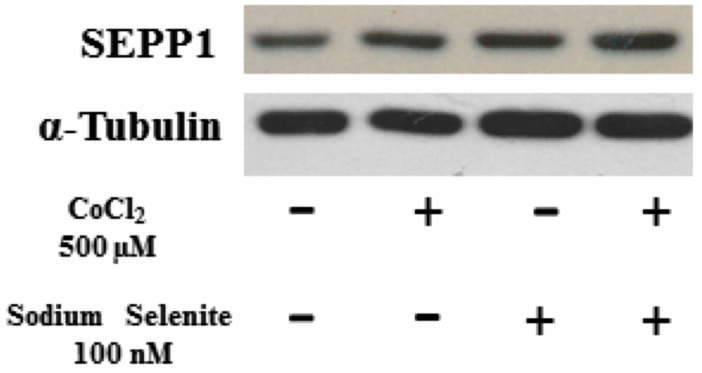
Effects of Na_2_SeO_3_ and CoCl_2_ on SEPP1 expression levels. Western blot analysis detected the protein expression of SEPP1 after incubation of cells with Na_2_SeO_3_ (100 nM) for 24 h and with CoCl_2_ (500 µM) alone or pre-treated with Na_2_SeO_3_ (100 nM) for 24 h. α-tubulin was used as a loading control.

Data from the literature indicates that for oxidative stress protection selenoproteins play an important role; moreover, the hypoxic conditions could represent a stimulus to adapt the expression of selenoproteins or selenium metabolism to the needs of cells [14]. So, in HK-2 cells, the expression of SEPP1 after treatment with CoCl_2_ alone or after pre-treatment with sodium selenite was evaluated in hypoxic conditions. As shown in Figure 5, incubation of cells with cobalt chloride alone revealed an enhanced expression of SEPP1 compared to untreated cells (*p* < 0.05), while in cells pre-treated with Sodium selenite before CoCl_2_ incubation an increase in SEPP1 expression was observed with respect to the control and with respect to CoCl_2_ alone.

## 4. Discussion

In our study we reproduced an in vitro model of ischemia/reperfusion (I/R) injury to explore the potential protective effect of selenium on damaged tubular cells and to assess the expression of Selenoprotein-p1 (SEPP1). We selected HK-2 cells due to their crucial role in renal reabsorption, which requires high ATP levels, via the activation of the mitochondrial oxidative phosphorylation system, resulting in the generation of elevated levels of reactive oxygen species [28,29].

Furthermore, SEPP1 binds megalin, a lipoprotein receptor with proximal tubule epithelium localization, demonstrating that kidney selenium homeostasis is mediated at the level of proximal tubule [31].

Another selenium transporter, known as selenium-binding protein 1 (SBP1), has already been shown to be a reliable and sensitive biomarker for early detection of AKI insults. Lee et al. identified SBP1 as a biomarker for detecting mercury-induced nephrotoxicity through proteomic analysis [29], while Kim et al. confirmed its utility in an experimental AKI model induced by cisplatin in animal models [30].

### 4.1. Selenium’s Antioxidant Properties

In our study, pre-treatment with sodium selenite significantly enhanced cell growth and reduced mortality both before and particularly after hypoxic induction. Selenium is well known in the literature for its protective properties on cellular and molecular pathways. It is a necessary component of the glutathione peroxidase (GPx)/reductase system [32], a critical cellular antioxidant defense system crucial for maintaining cellular redox balance [33,34,35]. In particular, it acts to neutralize reactive oxygen and nitrogen species in different organs [10]. Hypoxic conditions lead to increased reactive oxygen species (ROS) production, key indicators of oxidative stress which adversely affect tubules and glomeruli. ROS are produced by mitochondrial, cytosolic, and membrane enzymes and are highly reactive, leading to protein alterations, DNA damage, cellular senescence, and apoptosis [3]. ROS accumulation in tissues is probably the origin, but also the effect, of apoptotic cell death [3]. In our case, the pre-treatment with selenium had a positive effect; specifically, we observed a reduced release of ROS after selenium supplementation with respect to cells injured by R/I and not-treated cells.

Several studies on patients undergoing cardio-surgery operations have linked low selenium levels with increased oxidative stress. In cardiac patients, the usefulness of selenium administration in perioperative phases is controversial. Stoppe et al. [36], in a prospective observational study, measured selenium levels after the completion of cardiac surgery with CPB. Selenium concentration measured one hour after intervention was independently associated with the development of multi-organ failure and correlated with perioperative inflammation and intensive care unit length of stay. In the same paper, they were unable to demonstrate similar correlations with the pre-operative selenium status. Same authors in another study supplemented patients undergoing elective cardiac surgery with sodium selenite ev daily during their intensive care unit stay, observing increased activity of glutathione peroxidase (GPX) [37]. However, conflicting results arose from a multicenter study across Canada and Germany, which administered high doses of sodium selenite to cardio-surgery patients, showing no reduction in mortality and morbidity compared to a placebo [38].

### 4.2. SEPP1 Expression

In a recent paper, we established SEPP1 as an early and promising biomarker for acute kidney injury in patients undergoing major cardio-surgery operation. SEPP1 increased particularly between the 4th and 8th hours after CPB and with a strict relation with ischemia duration [9]. In the same paper, we were unable to demonstrate if this plasmatic protein, mainly of hepatic origin, accumulates due to decreased filtration by damaged glomeruli or if renal tubules augment the expression of SEPP1 as a defeat mechanism [9]. However, in our present experiments, we demonstrated that tubular cells injured by hypoxia indeed upregulate SEPP1 expression. Our findings do not exclude that, in vivo, acute kidney injury could lead to the accumulation of SEPP1 also because of a reduced filtration, but in light of the augmented renal production in vitro we hypothesize it could represent a defeat mechanism to increase the supply of selenium to cells. In the literature, SEPP1 stands as a pivotal player in selenium metabolism and antioxidant defense mechanisms within the human body [22,39]. While initially recognized for its role in maintaining selenium homeostasis, recent clinical investigations have shed light on its broader implications in health and disease. Gharipour et al., in a double-blind, placebo-controlled, randomized clinical trial in patients with coronary artery disease and metabolic syndrome, administered selenium tablets or a placebo. Although plasmatic levels of selenium did not change considerably, significant differences in SEPP1 levels were observed in subjects with metabolic syndrome. They suggested that an elevated level of SEPP1 was associated with a reduced risk of metabolic syndrome and a significant increase in SEPP1 could result in the elimination of this syndrome [40].

### 4.3. Limits of the Study

Our study needs further in vivo studies to validate the observed effects of sodium selenite supplementation on renal injury and SEPP1 expression. Among the limits of the study there is the use of an in vitro model using HK-2 cells, which may not fully recapitulate the complexity of in vivo renal physiology. Moreover, in the study we used a specific concentration of sodium selenite (100 nM), which may not represent the range of concentrations encountered in vivo and might limit the generalizability of the findings. Another limit of the study could be the short duration of the treatment with sodium selenite and CoCl_2_, which was limited to 24 h, but it may not reflect the chronic exposure observed in clinical scenarios. While sodium selenite supplementation showed beneficial effects, it could be important to consider potential off-target effects or interactions with other cellular pathways that were not investigated in our study.

## 5. Conclusions

In conclusion, our data proved our pivotal hypothesis that selenium supply administered in the form of sodium selenite has an overall protective effect on cells under hypoxic conditions. It has a strong impact on cell proliferation and viability, reducing cell mortality induced in vitro by CoCl_2_. Our results indicate also that cell morphology alterations induced by CoCl_2_ are less evident if cells are pre-treated with sodium selenite, thus stressing the idea that sodium selenite interferes with the structural and functional changes occurring in renal tubular cells in hypoxic conditions. Moreover, ROS production is reduced in the presence of sodium selenite, thus leading to the assumption that sodium selenite has an overall antioxidant role. Based on this hypothesis, an exogenous sodium selenite supplementation could be a therapeutic means of protection from acute kidney injury in the future. The expression and release of SEPP1 may be considered as a new promising biomarker of acute renal damage; therefore, in the future the possibility of measuring SEPP1 could be a tool to recognize the early onset of acute kidney injury. New studies, especially in vivo, should be performed to confirm our findings in view of new diagnostic–therapeutic frontiers. In this setting, omics technologies could offer a promising future for understanding selenium’s metabolic pathways, paving the way for targeted interventions and personalized approaches to selenium supplementation.

## Data Availability

Data are contained within the article.
